# Recent Analytical Method for Detection of Chemical Adulterants in Herbal Medicine

**DOI:** 10.3390/molecules26216606

**Published:** 2021-10-31

**Authors:** Rimadani Pratiwi, Ratu Hanifa Fayza Dipadharma, Ishmat Jati Prayugo, Olivia Angelina Layandro

**Affiliations:** 1 Department of Pharmaceutical Analysis and Medicinal Chemistry, Faculty of Pharmacy, Universitas Padjadjaran, Bandung 45363, Indonesia; ratu18005@mail.unpad.ac.id (R.H.F.D.); ishmat18001@mail.unpad.ac.id (I.J.P.); olivia18001@mail.unpad.ac.id (O.A.L.); 2 Drug Development Study Center, Faculty of Pharmacy, Universitas Padjadjaran, Bandung 45363, Indonesia

**Keywords:** adulterated drug, undeclared synthetic drug, herbal medicine, analysis, analytical method

## Abstract

Herbal medicine has become popular in recent years as an alternative medicine. The problem arises when herbal medicines contain an undeclared synthetic drug that is illegally added, since it is a natural product that does not contain any chemical drugs due to the potential cause of harmful effects. Supervision of herbal medicines is important to ensure that these herbal medicines are still safe to use. Thus, developing a reliable analytical technique for the determination of adulterated drugs in herbal medicine is gaining interest. This review aims to provide a recent analytical method that has been used within the past 5 years (2016–2021) for the determination of chemical adulterants in herbal medicine.

## 1. Introduction

Herbal medicines are widely used to treat diseases in many countries, as there are still many medicines which are reported to have severe side effects [1]. Herbal medicines are naturally occurring, plant-derived substances, containing phytochemical compounds used for treatment or medicinal purposes [2]. Since the market of herbal medicine is increasing every year, to date there have still been reports that found chemical adulterants in herbal medicines, thereby containing an undeclared synthetic drug. In Indonesia, in 2020, The Food and Drug Administration issued a press release regarding herbal medicine that contains undeclared synthetic drugs [3]. Based on their regulation, herbal medicine should not contain synthetic chemicals or medicinal isolation results. Some examples of adulterated herbal medicines are undeclared ingredients, such as sildenafil in the herbal extract [4], sibutramine phenolphthalein in slimming, dietary capsule supplements [5], and dexamethasone and prednisolone in herbal medicine pellets [6]. Adulteration with synthetic drugs can be life-threatening, especially when those medications cause potential interactions or can cause other medical conditions. Therefore, detecting the presence of undeclared synthetic drugs in herbal medicine is important. 

Many papers have reviewed this issue. Jamshed Haneef et al. in 2013 reviewed the analytical methods for the detection of undeclared synthetic drugs in traditional herbal medicines. They reported various analytical approaches for the detection of synthetic adulterants in herbal medicine including high-performance liquid chromatography (HPLC) with ultraviolet (UV) and diode array detectors (DAD), ion mobility spectrometry (IMS), high-performance, thin-layer chromatography (HPTLC), liquid chromatography-tandem mass spectrometry (LC-MS/MS), nuclear magnetic resonance (NMR) analysis, and hyphenated-mass spectrometric techniques [7]. Jacob Calahan et al. in 2016 reviewed the chemical adulterants in botanical dietary supplements from 1990 to 2015, and also the various analytical techniques used for their detection, including mass spectrometry-based techniques (MS), capillary electrophoresis (CE), hyphenated techniques, and thin-layered-based analytical techniques (TLC) [8]. 

The current review focuses on providing an overview of the recently available analytical technique used for the detection of chemical synthetic drugs illegally added in herbal medicines. The paper was selected based on the topic of analysis of adulterated or undeclared synthetic drugs in herbal medicines or natural products and published within the past 5 years (2016–2021). The results show that the analytical method has been developed; we found that chromatographic-based techniques and spectroscopy-based techniques are still widely used for the detection of chemical adulterants. In addition, another technique has been developed and modified for determining possible adulteration, such as UPLC combined with QTOF-MS/MS [9], infrared spectrophotometry combined with partial least square (PLS) [10] or a combination with multivariate calibration of stepwise multiple linear regression (SMLR) [11], TLC-SERS (surface-enhanced Raman spectroscopy) and densitometry [4,12]. The analytical devices, such as paper-based or polymer-based analytical devices [6,13], which offer a rapid and simple tool for detection, are also reviewed.

## 2. Chemical Adulterant in Herbal Medicine

To date, there have been many reports of adulterated herbal medicine and dietary supplements that claimed to be all-natural, when, in fact, they contained an undeclared synthetic drug to enhance their therapeutic effect. For example, the herbal drug for weight gain or weight loss purpose, containing sibutramine, phenolphthalein, orlistat, lorcaserin, fluoxetine, sildenafil, amfepramone [5,12,14,15,16,17,18], caffeine, trimethoxyamphetamine, vitamin E, tramadol, fluoxetine, rizatriptan, venlafaxine and methadone [18,19,20]; the sexual enhancer, containing sildenafil, tadalafil, aildenafil, sulfoaildenafil, and vardenafil [4,9,11,21]; and the pain reliever, containing paracetamol, dexamethasone, and prednisolone [6,10,22].

## 3. Chromatographic Method

The chromatographic method is still widely used for the detection of adulterated drugs because it has a high separation capacity utilizing a complex mixture, and can simultaneously detect multi-drug components in a sample. There are a variety of chromatography methods that have been used, such as thin-layer chromatography, liquid chromatography, and gas chromatography. Currently, most of the recent chromatography methods are embedded and combined with detectors, such as mass spectroscopy, Raman spectroscopy, and other detectors. Table 1 provides a list of the chromatographic methods that have been applied to detect the undeclared synthetic compounds of herbal medicines.

### 3.1. Thin Layer Chromatography

Thin Layer Chromatography (TLC) is one of the analysis methods performed through separation by chromatography. This method can be used for qualitative and quantitative analysis to identify samples of herbal medicine. This method is simple, fast, and the operating costs are inexpensive. Hence, it can be used in small laboratories to control adulterated drugs in herbal medicines. Despite the benefit of TLC, the selectivity of TLC is not sufficient for confirmation of illegal adulteration. The selectivity and sensitivity of TLC can be improved by selecting an appropriate detection for analysis. A recent study conducted by Minh et al. (2019) developed a thin layer chromatography method by combining TLC with Raman spectroscopy to increase the selectivity and sensitivity of the analysis [4]. Raman spectroscopy, especially surface-enhanced Raman spectroscopy (SERS), is a highly specific analytical technique that can be effectively used for qualitative analysis and chemical and physical structure elucidation [27]. Nonetheless, use of SERS is quite challenging when detecting analytes in the complex matrix, such as herbal products, which could hinder the SERS’ measurement. Combining TLC with SERS is a recent solution to detecting the undeclared synthetic compound in the complex matrix. Separation in TLC will minimize the influence of the complex matrix on the SERS measurement, meanwhile SERS will improve the selectivity and sensitivity of TLC detection.

In the research by Minh et al. [4], the TLC method was used to separate sildenafil as the analyte, from the similar and most commonly used PDE-5-inhibitor, which is tadalafil and vardenafil, as well as from another compound of complex herbal ingredients. The 5 µL of sample solution was sprayed on an aluminum TLC plate using an adjusted mobile phase of ethyl acetate-isopropanol-25% ammonia (45:5:2.6, *v/v/v*), then the spot was compared with the standard and the Rf calculated. Sildenafil, which had been separated by TLC, is then detected using Raman spectroscopy to determine the peak characteristics, including vibration, frequency, and intermolecular bonds. Surface-enhanced Raman spectroscopy amplifies Raman signals from molecules with much higher scattering efficiencies when adsorbed on metal colloidal nanoparticles or rough metal surfaces [28]. They used a silver nanoparticles (AgNPs) colloid as an enhancer to analyze sildenafil, discovering that the morphology had been characterized previously by UV-Vis Spectroscopy and transmission electron microscopy (TEM), resulting in a small nano and uniform size. The characteristic of AgNPs affects the formation of hot spots in thin layer chromatography that would result in a better enhancement of SERS. The concentration of silver nanoparticle colloids should be great enough to form a layer on the substrate particle to make a hot spot, but it also should not increase above the level required to form a monolayer. A higher concentration level of AgNPs will accumulate on the substrate, leading to SERS signal reduction. To ensure TLC-SERS results, all the real samples were analyzed by LC-MS/MS. The results acquired by LC-MS/MS are compatible with TLC-SERS results. The TLC-SERS method shows that the limit of detection of sildenafil was 10 μg/mL (2 ng/spot), and the concentration of the sildenafil sample ranged from 0.02 to 0.2 mg/mL.

Other than being coupled with SERS, the TLC method had also been developed with densitometric analysis, called TLC-densitometric. Densitometry is an instrumental analytical method based on the interaction of electromagnetic radiation with the analyte, which is a spot or stain on the TLC plate, for quantification purposes. The interaction of electromagnetic radiation with the stain on the TLC plate is determined to be the adsorption, transmission, or reflection of fluorine fluorescence, or the extinction of fluorine fluorescence from the original radiation [29]. A determination of sibutramine as an adulterated drug in herbal medicines using TLC-densitometry was conducted by Hayun et al. (2016) [12]. The LOD values obtained were at 217.5 ng and the LOQ values were 724.9 ng/spot. When compared with the concentration of the drug used, which is 0.50–5.00μg/spot, this method can be said to be analytically sensitive. Besides the LOD and LOQ value, it has an acceptable relative standard deviation, which were all less than 2%. The average recovery percentage is 99.70 ± 1.22, which is also acceptable. By these validation parameters, it was shown that TLC-densitometry method has a high accuracy and precision. Compared to TLC-SERS, TLC-densitometry is simpler and less consuming time. Nevertheless, the TLC-SERS have a better LOD, meaning that the method is more sensitive for measuring very small amounts of analyte. 

### 3.2. Liquid Chromatography

Another widely used chromatographic method is high-performance liquid chromatographic (HPLC), which can also be used for the separation of various components in the mixture. The separation principle of HPLC is based on the distribution of the analyte between eluent as a mobile phase and the packing material of the column as a stationary phase with high pressure through a column pump. The use of HPLC can be combined with various detectors, such as ultraviolet detectors (UV) and mass spectroscopy (MS). HPLC-UV analysis techniques can provide sensitive and reproducible analytical results that have a fast analysis time, a low sample requirement, high accuracy, and precision to determine simultaneous compounds in the sample [30]. An example of undeclared synthetic drug analysis on herbal products using HPLC-UV was conducted by Hemdan et al. (2018) [23] to analyze sibutramine, sildenafil, and phenolphthalein. Separation was achieved by the Inertsil C18 analytical column (4.6 × 100 mm with 5 μm particle size) with the mobile phase used for detection of sibutramine, and phenolphthalein was the potassium dihydrogen orthophosphate buffer (adjusted to pH 3 using o-phosphoric acid) and acetonitrile (40/60 *v*/*v*) with UV detection at 223 nm, while for SLD it was an acetonitrile–potassium hydrogen phosphate buffer (pH 3.2) adjusted using o-phosphoric acid (50/50 *v*/*v*) with detection at 230 nm. Analysis was performed at a flow rate of 1 mL/min. The result shows good agreement with HPLC-PDA and MS/MS.

Aside from being combined with a UV detector, HPLC can also be coupled with a tandem mass spectrometry (MS/MS). An example of the specification and validation of HPLC-MS/MS was conducted by Lawati et al. (2017) [21] to determine the adulteration of sildenafil, tadalafil, and vardenafil hydrochloride. The mobile phase of this method consisted of 0.1% formic acid in water (A) and 1% formic acid in 15% acetonitrile and 85% methanol (B). It was performed with gradient elution: 0–2 min 5% B, 2–4 min 5% B, 4–7 min 40% B, 7–14 min 65% B, 14–18 min 90% B, 18–23 min 90% B, 23–23.5 min 5% B, and 23.5–27 min 5% B to equilibrate for the next injection; the total run time was 27 min and the flow rate was 0.3 mL min^−1^. The main principle of mass spectrometry (MS) is to generate ions from either inorganic or organic compounds then separate these ions by their mass-to-charge ratio (*m*/*z*) and to detect them qualitatively and quantitatively by their respective *m*/*z* and abundance. Tandem mass spectrometry combines two mass analyzers in a single instrument to increase their abilities to analyze chemical samples.

Another liquid chromatography technique that can be used to detect adulteration of drugs is the ultra-high performance liquid chromatography (UPLC). UPLC operates at higher pressures (15,000 psi) and allows for lower particle sizes in columns, compared to HPLC that operates at lower pressures (max < 6000 psi). Both UPLC and HPLC have a similar accuracy and precision, however, UPLC has a better resolution, sensitivity, decreases in solvent consumption, and improves the quality of data [31]. UPLC is commonly combined with another detector, such as QTOF-MS or QTOF-MS/MS. QTOF-MS is a hyphenated analytical technique that combines the benefits of two different mass analyzers, namely the time of flight and the quadrupole mass analyzer. This method combined the analyzers by utilizing the high compound fragmentation efficiency of quadrupole technology with the rapid analysis speed and high mass resolution capability of time-of-flight. The Q-TOF MS uses a quadrupole (four parallel rods arranged in a square formation), a collision cell, and a time-of-flight unit to produce spectra. The first quadrupole (Q1) is capable of operating as a mass filter for the selection of specific ions based on their mass-to-charge ratio (*m*/*z*), or in radio frequency (RF) only mode where all ions are transmitted through the quadrupole. The second quadrupole (Q2) acts as a collision cell where ions are bombarded by neutral gas molecules, such as nitrogen or argon, resulting in the fragmentation of the ions. After leaving the quadrupole, ions are reaccelerated into the ion modulator region of the time-of-flight analyzer, where they are pulsed by an electric field and accelerated orthogonally to their original direction. All ions, having acquired the same kinetic energy, now enter the flight tube, which is a field-free drift region where mass separation occurs. Ions exhibiting a lighter mass will have a shorter time of flight, whereas heavier ions will take longer to traverse the flight path towards the detector [32].

A study conducted by Yu et al. (2016) [24] has successfully confirmed the adulteration of caffeine, chlorpheniramine, piroxicam, betamethasone, and oxethazaine of herbal capsule products, by using UPLC-QTOF-MS. The stationary phase used in this method was the T3 column (1.8 μm, 2.1 × 100 mm) and the column temperature was set at 45 °C. The flow rate was kept at 0.6 mL/min. The mobile phase consisted of water with 0.1% formic acid (A), and acetonitrile (B). The chromatographic separation was achieved by gradient elution from 1% B to 70% B in 26 min with an additional 2 min of re-equilibration. The total run time was 28 min, and the injection volume was 2 μL. The confirmation of 5 undeclared synthetic drugs was performed through the comparison of retention time, peak, and fragmentation pattern between the sample and standard. Chromatogram of chlorphenamine, oxethazaine, piroxicam, and caffeine are the major sample peaks, which implies that adulterants are present at higher concentrations than other natural compounds in the sample.

Another research that used the UPLC-QTOF mass spectrometry method was performed by Wang et al. (2018) [9]. Instead of using a single mass spectrometry, it used tandem mass spectrometry (MS/MS) coupled with 2 mass analyzers by using a collision cell, for improving the specificity of the mass spectrometer. It was used to detect sildenafil, tadalafil, aildenafil, and sulfoaildenafil in Chinese traditional patent medicines. Chromatographic separation was performed on an SB-C18 RRHD column of Agilent (100 mm × 3.0 mm, 1.8 mm). A binary mobile solvent was used: mobile solvent A was 5 mmol/L ammonium acetate solution (adjusted pH to 3.4 with acetic acid), and mobile solvent B was acetonitrile. The mobile phase was delivered at a flow rate of 0.4 mL/min with a gradient elution profile. The gradient began at 25% B for 2 min, and then linearly ramped to 55% B within 11 min, then ramped to 90% B in 1 min and held at 90% B for 2.0 min, then the column was re-equilibrated at 25% B for 2 min before the next injection. The autosampler tray temperature was set to 15 °C, while the column temperature was 40 °C and the injection volume was 5 mL. From the validation method, it can be concluded that UPLC-QTOF-MS/MS has a high sensitivity as the LOD and LOQ values are 0.002–0.1 mcg/g and 0.005–0.25 mcg/g, respectively. It was also considered as a high precision method based on the acceptable % recovery values, which were 82.5%–103.6%.

Jin et al. (2017) [25] have also developed a UPLC-MS/MS method with graphene as the sorbent for developing a microscale solid-phase extraction (SPE) using the pipette tip as a cartridge, namely Gtip. Graphene has a unique two-dimensional double-sided polyaromatic scaffold with a high specific surface area. The π-π electrostatic stacking property endows graphene with a strong affinity for carbon-based ring structures, making it a promising adsorptional material with high loading capacity. The Gtip SPE and UPLC–MS/MS method was developed for the quantitative analysis of trace levels of synthetic adulterants in slimming supplements. In this study, the liquid slimming supplement extract was aspirated into the conditioned Gtip and dispensed back to the sample tube. The eluate was dispensed as waste. After washing with 5% aqueous ACN, the Gtip was loaded on a vacuum manifold and dried under vacuum for 5 min. Finally, the adulterated drugs were eluted from the Gtip with 200 µL 5% ammonium hydroxide (25%) in ACN as a basic condition or 0.5% formic acid in ACN as an acidic condition by 10 repeated aspirating/dispensing cycles. The eluate was filtered through a membrane filter, then analyzed by LC-MS/MS. The Gtip showed efficient and reliable analytical performance in the preconcentration and enrichment of fenfluramine, phenolphthalein, bumetanide, and sibutramine. When compared with other commercial sorbents, such as C18 and HLB, graphene was more effective than these sorbents for Gtip SPE of trace fenfluramine, phenolphthalein, bumetanide, and sibutramine under similar conditions. The overall method showed high extraction efficiency, good specificity, accuracy, reproducibility, and sensitivity by the low levels of LOD and LOQ, low values of RSD, and also a high percentage of the recovery.

### 3.3. Gas Chromatography

Besides LC and TLC, another method that can be used to detect the adulteration of herbal medicines is gas chromatography. It is a sensitive, reproducible, accurate, and has a lower cost compared to HPLC but is rarely used in comparison to TLC and LC because it requires additional pretreatment to achieve high thermal stability and has a volatile compound [30]. The principle of LC and GC are almost similar, the only difference is that LC uses a solvent as its mobile phase, meanwhile, GC uses inert gases in the same capacity. The main characteristic that should be considered to analyze using gas chromatography is the volatility and thermal stability of the substances. Nitrogen (N2), hydrogen (H2), and helium (He) are three gases that are commonly used as carriers in gas chromatography (GC). Among those gases, the most commonly used carrier is helium. Helium is naturally found in gases and radioactive decay and is relatively rare in the atmosphere [27].

A recent study conducted by Lin et al. (2018) [26] uses hydrogen as an alternative gas carrier, in anticipation of a potential helium-shortage crisis, limited supply, and an expensive price in the future. Hydrogen offers some benefits for chromatography, including increased speed, lower temperature separations, longer column life, fewer environmental concerns, and greater availability. Gas chromatography is commonly coupled with mass spectrometry (MS) as a detector. The MS breaks each separate compound coming from the GC into ionized fragments, using a high-energy beam of electrons that are passed through the sample molecule to produce electrically charged particles or ions. Each charged fragment will have a certain mass. The mass of the fragment divided by the charge is called the mass-to-charge ratio (*m*/*z*). The fragments then go through a process of acceleration and deflection whilst traveling through a short tunnel and being exposed to a magnetic field. They eventually hit a detection plate at the end of the tunnel, where the mass-to-charge ratio (*m*/*z*) and relative abundance are calculated [33]. A compound is analyzed by GC-MS, not only by comparing its retention time to a standard (GC) but also by using its mass spectrum. As shown from Table 1, the LOD of this method is 10 to 1000 μg/g, which means that it has a good sensitivity to detect a low level of an analyte. Compared to LC and TLC, the preparation of GC samples is slightly more difficult because it requires a derivatization step preparation to convert them into the more volatile compound.

The chromatographic method offers various advantages for detecting adulterated drugs in herbal medicines, including selectivity, sensitivity, and wide applicability to detect various drugs. Therefore, this method is still commonly used for routine analysis in laboratories. However, this method is relatively high cost and the instrumentations are large, making chromatography unsuitable for on-site analysis.

## 4. Spectroscopic Method

A spectroscopic method is one of the widely used methods for detecting a component on a complex matrix, including adulterated drugs in herbal medicine. The spectroscopic methods that are commonly used include infrared spectroscopy, mass spectrometry (MS), and NMR spectrometry. Previous research that has been conducted to analyze synthetic compounds in herbal medicines is shown in Table 2.

### 4.1. Infrared Spectroscopy

Infrared (IR) spectroscopy is the simplest, most rapid, and non-destructive analytical method without any previous sample pre-treatment. Moreover, when no sample pre-treatment is required, it doesn’t need additional reagent during the analytical step. Hence, potentially harmful reagents are avoided, providing benefits for the environment and being cost-effective regarding chemical waste [10,11]. IR spectroscopy is used to determine structures and functional groups of compounds and identify them based on the absorption by a molecule of a particular type of light, in the IR region of the electromagnetic spectrum. Each chemically distinct molecule will have a different absorption pattern made up of the number and different types of bonds present, and the presence of different functional groups [34]. IR spectroscopy has been developed into the latest generation of IR, named Fourier-transform infrared spectroscopy. Fourier-transform infrared spectroscopy requires a mathematical process called Fourier transform to convert the raw data into the actual spectrum. The major difference between an FTIR spectrometer and a dispersive IR spectrometer is the Michelson interferometer. The Michelson interferometer, which is the core of FTIR spectrometers, is used to split one beam of light into two, so that the paths of the two beams are different. Then the Michelson interferometer recombines the two beams and conducts them into the detector where the difference of the intensity of these two beams is measured as a function of the difference of the paths [35].

An example of a study that used conventional IR and FTIR are shown in Table 2. Research conducted by Nugroho and Ritonga (2018) [10] determined the adulteration of dexamethasone in a traditional herbal medicine (THM) painkiller for joint pain, using infrared spectroscopy that combined with the partial least square (PLS). Adulteration of the undeclared synthetic drug caused complex spectra and the overlapping of absorption signals of various substances, which typically makes it difficult to interpret the spectra of the adulterated samples through use of an IR spectroscopy. Therefore, it is combined with the partial least square (PLS) method to separate spectra of the analyte (dexamethasone) from the spectra of an authentic traditional herbal medicine. Using the PLS method, maximum chemical information could be obtained from spectral data by permitting the selection of wavenumbers in complex spectra and linking changes in spectra to changes at various component levels simultaneously, by calculating the contribution of other spectra that can interfere with the spectrum. The PRESS and RMSECV values obtained as the result of the cross-validation model selection for dexamethasone in traditional herbal medicine painkillers for joint pain were 0.0022721 and 0.02902, respectively. Meanwhile, the RMSEC values obtained were 0.009455. This low value of RMSEC, RMSECV, and PRESS indicated the high accuracy and precision of the analytical method.

Other research that used FTIR as an analytical method to detect sildenafil citrate in herbal aphrodisiacs has been performed by Nugroho et al. (2018) [11]. To quantify the levels of sildenafil citrate in herbal medicines, this FTIR method is combined with the multivariate calibration of stepwise multiple linear regression (SMLR). Stepwise linear regression is a method of regressing multiple variables while simultaneously removing those that aren’t important. The result of this method validation obtained 0.000310913 as RMSEC values and 0.0009191 as PRESS values. Besides being combined with SMLR, FTIR can also be hyphenated with PLS Discriminant Analysis (PLS-DA). A study using the combination of FTIR-PLS DA method was conducted by Walkowiak et al. (2019) [36] to detect the adulteration of kaempferol, rutin, or quercetin on the *Ginkgo biloba* supplement. PLS-DA provides a separation with minimal probability of false classification for test samples. The RMSEC and RMSECV values obtained were 0.393 and 0.570, respectively. By comparing the RMSEC, RMSECV, and PRESS values of the three methods using IR spectroscopy, it can be concluded that the FTIR-SMLR method has the best accuracy and precision due to the lowest values of RMSEC, RMSECV, and PRESS.

Two-dimensional correlation infrared spectroscopy has been developed for analysis. Two-dimensional correlation spectroscopy (2DCOS) was employed for the identification of the ephedrine and pseudoephedrine present in illegally adulterated slimming herbal products (SHPs) that have been studied by Miao et al. (2016) [37]. In 2DCOS, similarity or dissimilarity among variations of spectroscopic intensities, which are induced by applying an external perturbation to the sample, and are examined by constructing correlation spectra defined by two independent spectral variable axes. By spreading congested or overlapped peaks along the second dimension, apparent spectral resolution is enhanced, and interpretation of complex spectra is simplified. Further information about the sequence of dipole reorientation or conformational change can also be obtained. When the contents of the adulterants are low, 1% for instance, 2DCOS analysis of SHPs becomes rather challenging. To obtain meaningful 2DCOS data, it is necessary to use suitable pretreatments before carrying out the actual correlation analysis to further improve the resolution. The study by Miao et al. (2016) [38] used the second derivative (SD) pretreatment to identify trace amounts of ephedrine and pseudoephedrine (0.5–1% to 5% contents) in slimming herbal products by deconvoluting overlapping peaks and enhancing resolution. Thermal perturbation was applied to obtain dynamic spectra for 2D correlation analysis. SD was carried out on 1D-FTIR before 2D synchronous spectral analysis to further enhance the spectral resolution and reduce the limit of detection (LOD). This pretreatment results in an improvement of LOD values, which are <1%, thus indicating that this method has a high sensitivity.

### 4.2. Nuclear Magnetic Resonance (NMR) Spectroscopy

Nuclear magnetic resonance (NMR) spectroscopy can also be used as a method to determine adulterated herbal products. NMR spectroscopy is an analytical technique used to determine the content and purity of a sample, as well as its molecular structure, by taking advantage of the magnetic properties of certain nuclei. The basic principle behind NMR is that some nuclei exist in specific nuclear spin states when exposed to an external magnetic field. NMR uses a large magnet to probe the intrinsic spin properties of atomic nuclei. As is the case with all spectroscopies, NMR uses a component of electromagnetic radiation (radiofrequency waves) to promote transitions between nuclear energy levels (resonance) [38]. Recent research conducted by Wu et al. (2020) [5] has used the development of the NMR method, low-field ^1^H NMR spectra, to analyze sibutramine and phenolphthalein in slimming dietary supplements. Low-field (LF) NMR is an emerging technique based on the use of a new generation of compact NMR. It presents an opportunity to replace costlier or destructive methods while utilizing non-deuterated solvents. The lowest limit value resulting from this method was 3 mg/100 mg, as shown in Table 2. This value shows that it is considered a sensitive method, but less sensitive compared to other spectroscopic methods.

### 4.3. Mass Spectrometry (MS)

Mass spectrometry (MS) is an analytical technique that is used to measure the mass-to-charge ratio of ions. The results are typically presented as a mass spectrum, a plot of intensity as a function of the mass-to-charge ratio [33]. Compared to other methods, MS can be used to detect a wider range of compounds. As shown in Table 2, a study by Hu et al. (2016) [39], using WT-ESI-MS, could analyze more than 5 undeclared drugs (melatonin, doxepin, diazepam, chlorpheniramine, zopiclone, nitrazepam, zaleplon, alprazolam, clonazepam, and chlordiazepoxide) from an herbal dietary supplement. wooden-tip ESI-MS (WT-ESI-MS) is a technique that could be used for the direct analysis of raw samples. This technique makes use of readily available, economical, and disposable wooden toothpicks, which can be directly compatible with commercially available nano ESI ion sources, for sampling and ionization. The slim and hard wooden tips are very convenient for sampling, and the technique could be used for the analysis of samples of various forms. By using this method, the LOD values that were obtained were 0,1 mg/g, which signifies a good sensitivity analytical method.

Other than being combined with wooden-tip-ESI, MS can also be combined with fast-switching +/− HV tip-ESI. Research was conducted by Yao et al. (2019) [40] to analyze the adulteration of paracetamol, naproxen, sulfamethoxazole, diclofenac, and phenylbutazone in herbal dietary supplements. Electrospray ionization mass spectrometry (ESI-MS) was commonly performed to obtain accurate results, due to its desirable sensitivity and specificity. In most of the previous work on tip-ESI, the analytes were detected in either positive or negative ion mode. However, various synthetic drugs are alternatively amenable to either a positive or negative ion mode but simultaneously exist in adulterated herbal dietary supplements, posing a challenging task for simultaneous detection of positive and negative ions by MS. By using a fast-switching positive/negative high-voltage (+/− HV) that was applied to tip ESI-MS for the simultaneous screening of five synthetic drugs, the MS detection mode (+/−) was automatically switched, and, accordingly, the drugs can then efficiently be detect in positive/negative mode. The LOD values obtained from 5 synthetic adulterated drugs were all <0.1 ng/g. These LOD values indicate a high sensitivity of fast-switching +/− HV tip-ESI-MS, which is superior compared to WT-ESI-MS.

A recent study by Wang et al. (2020) [41] has also used MS, which was developed with ultrasonic extraction and nebulization in real-time, coupled with carbon fiber ionization (UEN/CFI-MS), to screen antidiabetic drugs, an antihypertensive drug, and hypolipidemic drug adulteration in herbal products. UEN/CFI is a pretreatment method used to ionize samples without adding auxiliary gas or a heating system. Compared with electrospray ionization (ESI), UEN/CFI has shown great compatibility with both polar and non-polar compounds. In ESI-MS, compounds with extremely low polarity, such as anthracene, are hard to detect, meanwhile, UEN/CFI exhibits no compromise for the detection of polar compounds. UEN and CFI were separated as two independent ionization sources. UEN was used as the ultrasonic extraction and nebulization device that acts to efficiently desorb the analytes from the sample. Then, the tiny droplets containing desorbed analytes were efficiently ionized by CFI-MS. During the research conducted by Wang et al. (2020), the length of carbon fiber was 0.8 mm and the voltage on the carbon fiber was ±3.0 kV, meanwhile, the capillary temperature of the mass spectrometer was controlled at 300 °C. The assisted solvent (methanol) was injected through a syringe pump at a flow rate of 5.0 mL/min. The UEN was placed directly above the tip of the carbon fiber with a vertical distance of 6.0 cm. The sample solution was placed directly in the center of the UEN for liquid samples, while solid samples were directly analyzed after adding extraction solvents. The analyte inside the sample was extracted and nebulized efficiently by UEN. Atomized sample droplets were ionized by the CFI ion source, then the ion signals of the sample were captured by the mass spectrometer. The LOD values obtained by UEN/CFI-MS were 2 mcg/g to 50 mcg/g, which indicates a high sensitivity. Furthermore, the RSD values were less than 15%, which shows that it has a decent analytical method precision.

Similarly to the chromatographic method, spectrophotometric is still widely used for routine analysis in laboratories. This method also offers good selectivity and sensitivity; however, this method is highly costly and usually must act in tandem with another method to improve the analytical performance.

## 5. Microfluidic Analytical Device

Numerous conventional instrumental analytical techniques are used for the determination of adulterated drugs in herbal medicines, such as the chromatographic-based and spectroscopic-based methods that were previously described. These techniques require expensive equipment, highly trained operators, and are only suitable for routine analysis in the laboratory. Recently, the utilization of microfluidic analytical devices for drug analysis has been developed. This device offers several advantages, such as simplicity, low cost, rapid analysis, portability, and low consumption of reagent and sample [42,43]. The first microfluidic paper-based analytical device (µPAD) was developed for point-of-care medical diagnostic [44] and is now being developed for bacterial, pesticides, organic molecules, metal, and drug analysis [45,46]. Various substrates have been used for fabricating microfluidic devices, including paper-based [47,48,49] and polymer [50].

**Figure 1:**
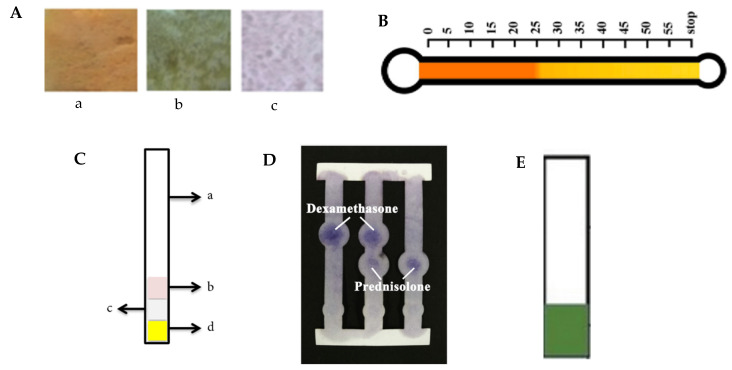
Visualization of the microfluidic analytical device for detection of undeclared synthetic drugs in herbal medicine. (**A**) Optical sensor membrane based on polymer poly-methyl methacrylate. a (PMMA-FeCl3 7.5% 7:3); b (PMMA- Folin–Ciocalteu 5% 6:4); c (PMMA-Nessler’s reagent 5% 6:4). Reprinted with permission from Ref. [22] (Copyright 2018, Hindawi). (**B**) Schematic of the distance-based paper device for the determination of sibutramine. Reprinted with permission from Ref. [58] (Copyright 2021, Elsevier). (**C**) Design of paper-based analytical device for allopurinol detection. a (support base); b (Whatman filter paper content Folin–Ciocalteu reagent); c (Whatman filter paper content p-DAB reagent); d (Whatman filter paper content tollens reagent). Reprinted with permission from Ref. [13] (Copyright 2019, Hindawi). (**D**) Visualization of a paper-based analytical device for dexamethasone and prednisolone detection. Reprinted with permission from Ref. [6] (Copyright 2019, Elsevier). (**E**) The dipstick for dexamethasone detection. Reprinted with permission from Ref. [59] (Copyright 2021, Mahidol University).

The microfluidic device was also developed in the determination of undeclared synthetic drugs in herbal medicine, as shown in Table 3. The visualization of all the microfluidic device that has been reviewed is shown in Figure 1. A study conducted by Pratiwi et al. (2018) [22] developed an optical sensor device based on polymer poly(methyl methacrylate) (PMMA) for paracetamol detection in herbal medicine. PMMA offers some benefits as a membrane material, due to its mechanical strength, chemical inertness, and high thermal stability [51]. A phase inversion method was applied for fabrication of the polymer membrane and colorimetric was chosen as a detection method. In this experiment, they vary the concentration of PMMA to 5%, 7.5%, and 10%, and each concentration was dissolved in the mixture with a colorimetric reagent, such as ferric chloride, Folin–Ciocalteu reagent, Nessler’s reagent, and Lieberman reagent. The mixture ratio of the solvent and reagent was 6:4, 7:3, and 8:2. The best results show on PMMA 7.5%-FeCl_3_ (7:3), PMMA 5%-Folin (6:4), and PMMA 5%-Nessler (6:4). The performance of this device, including its sensitivity, stability, and selectivity, is acceptable and shows good agreement with the spectrophotometry method. The visualization of the optical sensor membrane is shown in Figure 1A.

Other research develops a colorimetric paper-based analytical device for allopurinol detection in herbal medicine [13]. The study was conducted by using Whatman filter paper No. 1, No. 2, and No. 4 and Whatman chromatography as a substrate, and nine colorimetric reagents (dragendorf reagent, ferric chloride, Folin-Ciocalteu reagent, sodium nitroprusside, p-DAB reagent, Schiff reagent, potassium chlorate, tollens reagent, and sodium nitrite) based on the reaction with allopurinol. The result shows that only Folin-Ciocalteu, tollens, and p-DAB reagent can be applied to the paper. Folin-Ciocalteu will give a dark blue color when reacting with the allopurinol solution, meanwhile, tollens and p-DAB reagent give a silver and yellow color, respectively. The design of PAD following the design of universal pH indicator strip and PAD containing 3 of specific reagent, as shown in Figure 1C. The results show that there was no significant difference in the results of the varying papers, and the lowest measurable detection of PAD is 75 mg/mL. The device is selective due to having different color changes with another interfering compound. Application in the real sample shows good agreement with TLC and the spectrophotometry data in 75 mg/mL.

Colorimetric is one of the detection methods commonly used for PAD, due to its simplicity, high contrast when using paper as a substrate, and low-cost detection system [52]. However, this detection method lacks sensitivity and selectivity [53]. To improve the sensitivity and selectivity, electrochemical detection can be applied for PAD through the selection of the electrode material, measurement techniques, and detection scheme [46]. In addition, multiple drug compounds are electrochemical active, making this detection potential for direct detection [54]. Primpray et al. (2019) [6] developed the paper-based analytical device with electrochemical detection for the determination of dexamethasone and prednisolone in traditional medicine. They used Whatman SG81 silica-coated paper and a design that had three channels and two different bulb shapes in the bottom and upper areas, as shown in Figure 1D. The device was printed by a 3D printer with PLA filament polymer and printed as a 3D-printed cassette, providing two inlets for screen-printed carbon electrode (SPCE) insertion, together with a 3D printed cutter. The optimal mobile phase was 60% ethyl acetate in cyclohexane, and the limits of detection for dexamethasone and prednisolone were 3.59 and 11.98 µg/mL, respectively, whereas the limits of quantification were 6.00 and 20.02 µg/mL, respectively. Analysis in real samples was compared with the standard HPLC method and the result showed good correlation.

Another detection method that can be applied in PAD is distance-based detection. This detection measures the length of color change along the channel of paper and is recently widely applied as a device for metal analysis [55,56]. Distance-based detection offers a simplified technique for quantitative detection [57]. The distance-based paper analytical device for sibutramine detection in slimming products was developed by Karamahito et al. (2021) [58]. They used filter paper printed with thermometer-shaped and a ruler scale printed parallel along the channel, as shown in Figure 1B. Dragendorff’s reagent was used along the channel to form an orange-red precipitate of the sibutramine-tetraiodobismuthate complex. The length of the color change is proportional to the amount of sibutramine in the sample. The result shows that this device has LOQ 0.22 mmol/L and a precision of less than 4.4%RSD. The result is also in agreement at 95% confidence level with the gas chromatographic method. This device offers simple, low-cost, and instrument-free onsite analysis.

A simple and rapid analytical device for the detection of adulterated drugs in herbal medicine was also developed by Kuswandi et al. (2021) [59]. They developed a dipstick test for dexamethasone detection constructed by immobilization of FeCl_3_ and K_3_[Fe(CN)_6_] onto cellulose acetate film in acid conditions, as shown in Figure 1E. The cellulose acetate was activated by increasing the porosity of film and de-esterification. The reagent was immobilized in the detection zone of the film. The color change was green to blue in the presence of dexamethasone and captured using ImageJ for quantitative measurement. The results show the dipstick test has linearity in 0.5–75 μg/mL, and the LOD and LOQ were 0.422 μg/mL and 1.406 μg/mL, respectively. Application in the various sample shows a good agreement with UV spectrophotometric. This device offers an alternative tool for dexamethasone detection in herbal medicine.

The microfluidic analytical device can be an alternative instrument-free method for the detection of adulterated drugs in herbal medicine. This device is designed for simple, portable, and easy use for direct analysis. The performance of this method can be improved by selecting an appropriate detection for analysis.

## 6. Electrochemical Method

The utilization of the electrochemical method has a considerable attraction for drug analysis. Electrochemical detection offers improved selectivity, sensitivity, and can also be carried out using portable instruments [60]. The application of this method has been demonstrated by Freitas et al. (2018) [61] for sibutramine detection in a natural product. They used the square-wave voltammetric (SWV) method adapted to a portable batch-injection analysis (BIA), which provided a simple and portable fast screening and quantification of sibutramine in herbal products. The voltammetric system was performed using a PGSTAT 128N potentiostat/galvanostat and used a boron-doped diamond (BDD) as the working electrode. The sample was dissolved in 0.1 mol/L H_2_SO_4_ as an optimum supporting electrolyte. The result shows the recovery value was 105 to 113% with the relative standard deviation less than 3% and the LOD was 0.08 to 1.94 mg/L. This system claims to be able to screen 200 analyses without handling the electrodes of the BIA-SWV system, and is also suitable for determination up to 1% of sibutramine in the sample.

Other research conducted by Saichanapan et al. (2020) [62] uses porous graphene ink-modified electrodes on the glassy carbon electrode surface (PGr-ink/GCE) to improve sensitivity for the determination of sibutramine in slimming products. ATR-FTIR spectroscopy and SEM were applied for surface characterization, meanwhile cyclic voltammetry (CV) and electrochemical impedance spectroscopy (EIS) were applied to study the electrochemical adsorption behavior of the PGr-ink/GCE. The adsorption of sibutramine on PGr-ink/GCE was examined by square wave adsorptive stripping voltammetry (SWAdSV) to determine the concentration of sibutramine. The result showed that PGr-ink/GCE exhibited a sensitivity four times higher than bare GCE, and the limit of detection and quantification were 5 ng/mL and 15 ng/mL, respectively. In addition, this system shows good reproducibility and repeatability at less than 3.4% and RSD was 1.8 to 9.8%, respectively. The developed PGr-ink/GCE offers a simple, low detection limit, and a high sensitivity sibutramine sensor for detecting sibutramine in the sample.

Electrochemical detection can be used as another approach for analyzing adulterated drugs in herbal medicine, since the sensitivity and the selectivity of the assay can improve by selecting electrode material and measurement techniques. In addition, this technique can combine with a portable addition device, creating the possibility for on-site analysis.

## 7. Conclusions

Traditional herbal medicine has gained approval, especially in developing countries, as an alternative or complementary medicine. The safety of herbal products has become a concern due to the herbal products that have been found to contain undeclared synthetic drugs. Many regulations state that herbal medicines should not contain illegal drugs due to the side effects of uncontrolled consumption, such as headaches, nausea, insomnia, diarrhea until hematological abnormalities, mental depression, and even inducing a coma. Reliable analytical techniques are important for detecting adulterated drugs in herbal medicine to ensure the quality of herbal medicines and to protect human health. For routine analysis in the laboratory, the use the of instrument techniques, such as chromatography-based and spectroscopic-based with various detectors, or coupled with another detection method, is most likely. The instrumental technique provides excellent accuracy, precision, and sensitivity in the determination of adulterated drugs. These techniques required large instrumentation, making them inappropriate for on-site analysis. The instrumental-free analysis has been developed for portable analysis. The platform uses analytical devices based on paper, polymer, or film as a medium for the determination of drugs. This platform offers a simple, easy and low-cost alternative tool for on-site detection. The detection method can be selected based on the practical considerations and required analytical figures of merit. In addition, improving the analytical method is still necessary to provide an alternative method that can be adapted as required.

## Figures and Tables

**Table 1:** Various chromatographic methods for determining adulterated drugs in herbal medicine.

Analyte	Matrix	Method	Elution Type and Mobile Phase/Flow Rate	Column/Temperature	Detector	LOD/LOQ	% RSD/ Recovery	Ref.
Sildenafil	Capsule, granule, herbal extract claimed for treatment of erectile dysfunction	TLC-SERS	Mobile phase: ethyl acetate-isopropanol-25% ammonia (45:5:2.6, *v*/*v*/*v*)	Stationary phase: 20 × 10 cm aluminium TLC plate	Surface-enhanced Raman Spectroscopy (SERS)	LOD = 2 ng/spot	-	[4]
Sibutramine	Herbal slimming product	TLC-Densitometric	Mobile phase: toluene-diethylamine (10:0.3, *v*/*v*)	Stationary phase: aluminum TLC plates coated with silica gel 60 F254 with 250 μm thickness	Densitometric scanning at 227 nm with Camag^®^ TLC Scanner III	LOD = 217.5 ng LOQ = 724.9 ng/spot	Average recovery (%): 99.70 ± 1.22 RSD: <2%	[12]
Sibutramine phenolphthalein, sildenafil	Capsules (Sliming Bomb, Zotreem Plus, and Enjoy)	HPLC-UV	Mobile phase SIB and PPH: potassium dihydrogen orthophosphate buffer (adjusted to pH 3 using o-phosphoric acid)/acetonitrile (40/60 *v*/*v*) Mobile phase SLD: acetonitrile–potassium hydrogen phosphate buffer (pH 3.2) adjusted using o-phosphoric acid (50/50 *v/v*) Flow rate: 1 mL/min	Inertsil C18 Column (4.6 × 100 mm with 5 μm particle size)	UV at 223 nm for SIB dan PPH and UV at 230 for SLD	-	RSD = 1.926% (SIB), 1.779% (PPH), 1.709% (SLD) Recovery: 98.64 ± 1.151 (SIB), 98.78 ± 1.537 (PPH), 99.11 ± 1.814 (SLD)	[23]
Sildenafil, tadalafil, vardenafil hydrochloride	Capsule, cream, gel, etc. from 33 screened samples supplied by Public Authority of Customer Protection, Ministry of Health.	HPLC-MS-MS	Gradient elution. 0–2 min 5% B, 2–4 min 5% B, 4–7 min 40% B, 7–14 min 65% B, 14–18 min 90% B, 18–23 min 90% B, 23–23.5 min 5% B, and 23.5–27 min 5% B to equilibrate for the next injection (the total run time was 27 min). Mobile phase: A = 0.1% formic acid in water; B = 1% formic acid in 15% acetonitrile and 85% methanol flow rate: 0.3 mL min^−1^	Stationary phase: Poroshell 120 EC C18 column (3.0 ID × 100 mm length, 2.7 μm) (PC18) Temperature: 40 °C	diode array	-	Coefficient variance CV = < 4% Relative error RE = < 3%	[21]
Caffeine, chlorpheniramine, piroxicam, betamethasone, oxethazaine	Capsule *	UPLC-QTOF-MS	Mobile phase: water with 0.1% formic acid (A) and acetonitrile (B) Elution type: gradient elution from 1% B to 70% B in 26 min with additional 2 min re-equilibration Flow Rate: 0.6 mL/min	ACQUITY UPLC HSS T3 column (1.8 μm, 2.1 × 100 mm) Temperature: 45 °C	QTOF-MS with DIA as data acquisition	-	-	[24]
Sildenafil, tadalafil, aildenafil, sulfoaildenafil	Pellets, capsules, tablets, or oral liquid, claimed functions of aphrodisiac, enhancement of sexual performance, physical fatigue relief or immunity enhancement	UHPLCQ-TOF HRMS	Gradient elution: began at 25% B for 2 min, then linearly ramped to 55% B within 11 min, and then ramped to 90% B in 1 min, and held at 90% B for 2.0 min mobile phase: A = 5 mmol/L ammonium acetate (adjusted pH to 3.4 with acetic acid) B = acetonitrile. Flow rate: 0.4 mL/min	Column: Agilent SB-C18 RRHD column (100 mm × 3.0 mm, 1.8 mm) Temperature: 40 ℃.	QTOF-MS/MS	LOD = 0.002–0.1 µg/g LLOQ = 0.005–0.25 mcg/g	Recovery: 82.5–103.6%. RSD (Intra and inter-day): 0.4 to 13.6%.	[9]
Fenfluramine, phenolphthalein, bumetanide, and sibutramine	Slimming supplement: tea powder, capsule, tablets	Gtip SPE- UPLC–MS/MS	Gradient elution: initial 10% A, 4 min 50% A, 6 min 60% A, and 10 min 100% A. Total run time of 12 min. Mobile phase: Acetonitrile (A) and ultrapure water with 0.1% formic acid (B). flow rate: 0.40 mL/min	Stationary phase: Halo^TM^ fused-core C18 column (100 mm × 2.1 mm, 2.7 mcm) Temperature column 30 °C	MS/MS	LOD = 1.8 ng mL^−1^) LOQ = 5.6 ng mL^−1^	Recovery = 82.9–95.2% RSD = <7.3%	[25]
Amitriptyline acetaminophen, Ibuprofen, chlorzoxazone, sulfamethoxazole, tadalafil, and sildenafil	83 Traditional Chinese medicines and 40 food supplements samples	GC-MS	Hydrogen as a carrier gas; 1.0 mL/min	A silica capillary column, Agilent HP-5 MS (30 m × 0.25 mm i.d. 0.25 μm film thickness); temperature programming	MS electron ionization	LOD = 10 to 1000 μg/g.	-	[26]

* The detail herbal matrices available in the article.

**Table 2:** Spectrophotometric methods for determining adulterated drugs in herbal medicine.

Analyte	Matrix	Method	Max Wavelength or Frequency	LOD/LOQ	%RSD/Recovery	Reference
Dexamethasone	Herb claimed for joint painkiller *	Infrared spectroscopy combined with partial least square (PLS)	Wavenumbers 3646, 3642, 2461, 2453, 2432, 2406, 2229, 2209, 2197, 2097, 2092, 2064, 2059, 2047, 2026, 2009, 1969, and 1513 cm^−1^	-	Validation: R^2^ = −0.9988 RMSEC = 0.009455 PRESS = 0.0022721 RMSECV = 0.02902	[10]
Sildenafil	An herbal product claimed to enhance sexual activity *	FTIR-SMLR Stepwise multiple linear regression	wavenumbers 1791, 1692, 1691 and 971 cm^−1^	-	Validation: R^2^ = 1.00 RMSEC = 0.000310913 PRESS = 0.0009191	[11]
Sibutramine, phenolphtalein	Capsule, tablet, powder for weight-loss	Low-field ^1^H NMR spectroscopy	Frequency = 59.7 mHz for ^1^H	Lowes limit 3 mg/100 mg	-	[5]
Rutin, quercetin, kaempferol	Capsule and tablet containing *Ginkgo biloba*	FTIR-PLS DA	Wavelength = 900–1800 cm^−1^	-	Validation RMSEC = 0.393 RMSECV = 0.570	[36]
Ephedrin, pseudoephedrine	Slimming herbal product	2DCOS	Wavelength: 4000–400 cm^−1^	LOD = <1%	-	[37]
Melatonin, doxepin, diazepam, chlorpheniramine, zopiclone, nitrazepam, zaleplon, alprazolam, clonazepam and chlordiazepoxide	Herbal dietary supplements (pills, tablets, capsules, or soft-gel capsules)	WT-ESI-MS	Tranquilizer and aphrodisiac samples = 230 nm Weight loss samples = 225 nm or 265 nm.	LOD = 0.1 mg/g	-	[39]
Paracetamol, naproxen, sulfamethoxazole, diclofenac, and phenylbutazone	Tablet and capsules (anti rheumatism health care products)	Fast-switching +/− HV tip-ESI--MS	-	LOD: (<0.1 ng/g) PCM = 0.05 ng/g NPX 0.1 ng/g for NPX, 0.01 ng/g for SMZ, 0.01 ng/g for DCM, and 0.1 ng/g for PBZ	-	[40]
Nifedipine, nisoldipine, nicardipine, lercanidipine, felodipine, clevidipine, metformin hydrochloride, lovastatin, Simvastatin, gemfibrozil, and fenofibrate	Tablets, pills, granules, and capsules	UEN/CFI-MS	-	LOD = 2 mcg/g to 50 mcg/g	RSD = less than 15%	[41]

* The detailed herbal matrices available in the article.

**Table 3:** Various analytical devices for determining adulterated drugs in herbal medicine.

Analyte	Matrix	Method	Media	LOD/LOQ	%RSD/ Recovery	Reference
Paracetamol	Herbal medicine powder	Optical sensor membrane	Polymer poly(methyl methacrylate) (PMMA)	Lowest measurable: 2.55–4.01 mg/mL	-	[22]
Allopurinol	Herbal medicine powder	A paper-based analytical device using colorimetry	Whatman filter paper No. 1, 4, 6 and Whatman 1 chromatography	Lowest measurable: 75 mg/mL	-	[13]
Dexamethasone and Prednisolone	Herbal medicine pellets	Electrochemical paper-based analytical device	Whatman SG81 paper	LOD dexamethasone: 3.59 µg/mL prednisolone: 11.988 µg/mL LOQ Dexamethasone: 6.00 µg/mL prednisolone: 20.02 µg/mL	Dexamethasone: 83 to 108% (recovery) and 5.6 to 10.1% (Coefficient of variation) prednisolone: 88 to 134% (recovery) and 4.4 to 13.8% (coefficient of variation)	[6]
Sibutramine	Powder (weight loss product)	A distance-based paper device with colorimetry	Filter paper	LOQ 0.22 mmol/L	Less than 4.4%RSD	[58]
Dexamethasone	Powder (joint-paint killer product)	Dipstick test using sensing film	Cellulose acetate film	LOD: 0.422 μg/mL LOQ: 1.406 μg/mL	Recovery: 99.978–101.144% RSD: <0.196%	[59]
